# Luminescent Hybrid BPA.DA-NVP@Eu_2_L_3_ Materials: In Situ Synthesis, Spectroscopic, Thermal, and Mechanical Characterization

**DOI:** 10.3390/ma16196509

**Published:** 2023-09-30

**Authors:** Dmytro Vlasyuk, Renata Łyszczek, Beata Podkościelna, Andrzej Puszka, Zbigniew Hnatejko, Marek Stankevič, Halina Głuchowska

**Affiliations:** 1 Department of General and Coordination Chemistry and Crystallography, Faculty of Chemistry, Institute of Chemical Sciences, Maria Curie-Skłodowska University, M. C. Skłodowskiej Sq. 2, 20-031 Lublin, Poland; halina.gluchowska@mail.umcs.pl; 2 Department of Polymer Chemistry, Faculty of Chemistry, Institute of Chemical Sciences, Maria Curie-Skłodowska University, Gliniana 33, 20-614 Lublin, Poland; beata.podkoscielna@mail.umcs.pl (B.P.); andrzej.puszka@mail.umcs.pl (A.P.); 3 Department of Rare Earths, Faculty of Chemistry, Adam Mickiewicz University in Poznań, Uniwersytetu Poznańskiego 8, 61-614 Poznań, Poland; zbigniew.hnatejko@amu.edu.pl; 4 Department of Organic Chemistry, Faculty of Chemistry, Institute of Chemical Sciences, Marie Curie-Skłodowska University, Gliniana 33, 20-614 Lublin, Poland; marek.stankevic@mail.umcs.pl

**Keywords:** hybrid materials, polymeric matrix, metal complex, thermal properties, luminescence, mechanical properties, hardness, dynamic mechanical analysis

## Abstract

A series of homogeneous hybrid BPA.DA-NVP@Eu_2_L_3_ materials were obtained through an in situ approach where the luminescent dopant was formed at the molecular level with different contents (0.1; 0.2; 0.5; 1; and 2% by weight). A Europium(III) complex (Eu_2_L_3_) with quinoline-2,4-dicarboxylic acid was applied as a luminescence additive while a polymer matrix consisted of a combination of bisphenol A diacrylate (BPA.DA) and N-vinylpyrrolidone (NVP) monomers. Synthesis steps and the final materials were monitored by NMR and Fourier transform infrared spectroscopy (FTIR). The emission, excitation spectra, lifetime, and quantum yield measurements were applied for the determination of the photophysical characteristics. The thermal and mechanical properties of the obtained materials were tested via thermal analysis methods (TG/DTG/DSC and TG-FTIR) in air and nitrogen atmospheres, dynamic mechanical analysis (DMA), and hardness and bending measurements. Generally, even a small addition of the metal complex component causes changes in the thermal, mechanical, and luminescent properties. Hybrid materials with a greater europium complex content are characterized by a lower stiffness and hardness while the heterogeneity and the flexibility of the samples increase. A very small amount of an Eu_2_L_3_ admixture (0.1% wt.) in a hybrid material causes an emission in the red spectral range and the luminescence intensity was reached for the BPA-DA-NVP@1%Eu_2_L_3_ material. These materials may be potentially used in chemical sensing, security systems, and protective coatings against UV.

## 1. Introduction

From year to year, the tendency to develop and obtain new hybrid materials with specific properties is growing. This is due, among others, to the wide application spectrum of such materials and the high requirements for their properties, such as good thermal stability and mechanical strength. The combination of inorganic and organic components allows for obtaining hybrid materials with advanced properties that are widely used in modern fields, such as chemical sensors, optoelectronics, laser systems, environmental protection, medicine, etc. [1,2,3,4,5,6,7,8,9].

Polymer matrices play an important role in the process of construction of hybrid materials. They have a direct impact on such properties as flexibility, refractive index, conductivity, density, thermal stability, and mechanical strength. A properly selected polymer matrix will mainly determine the form and structure of the obtained material; therefore, before designing a new material, selecting and synthesizing the appropriate matrix is a very important stage. Widely used polymer matrices are PMMA, PVA, PLA, PAN, PBO, PA6, PDMS, PLGA, NVP, and BPA.DA, PSS, EGDMA, PEG, epoxy, cellulose, etc. Polymer matrices are mainly composed of synthetic molecules, monomers, and polymer-based materials, structurally distinguished as coatings and carriers, while compositionally, they consist of hydrogels, layer-by-layer (LbL) assemblies, polymer brushes, and block copolymer structures [10,11,12,13,14,15].

Inorganic compounds and metal complexes are utilized as functional additives for polymer matrices due to their good magnetic and optical properties, which, together with high chemical and thermal stability, allow for their use in various operating conditions. These additives are incorporated into the structure of the polymer matrix through various types of interactions (e.g., electrostatic attraction, hydrogen bonds, covalent bonds, Van der Waals forces) at the molecular level, allow for improving the properties of the pure matrix, and/or introducing additional functions, thus creating new materials [16,17,18,19,20,21].

The choice of the appropriate method for the synthesis of hybrid material is essential in the formation of a material with the desired properties. The dispersion and homogenization of monomers and functional additives in the polymeric hybrid materials are key factors influencing their mechano–physical properties. There are two possible main ways of combining the polymer–inorganic components: in situ or ex situ. The in situ method consists of the formation of the polymer part and dopant simultaneously during the formation of the hybrid material [22]. On the other hand, the ex situ method involves the formation of polymeric and inorganic components separately, and the hybrid material is created when they are combined [17,18,23,24,25,26,27]. Both of the presented general methods for the synthesis of hybrid materials have their advantages and disadvantages. The in situ method ensures a high level of homogenization and dispersion of material components which is the obvious benefit of such a method. Additionally, the formation of active dopant particles is often associated with direct bonding to the polymer backbone of the matrix through functional groups. In the ex situ method, the incorporation of a pre-synthesized dopant into the polymeric matrix can lead to inhomogeneity that negatively impacts on the material properties. Adding an admixture with a confirmed chemical composition is a positive aspect of this synthesis approach [24,26,28,29]. Hybrid material synthesis often involves a combination of these approaches that are tailored to the properties of the material’s components [1,2,13].

Lanthanide compounds, particularly europium(III) complexes, are commonly utilized as luminescent additives in multifunctional materials [2,30,31,32,33,34,35,36,37,38]. The low emission efficiency of lanthanide ions which is related to poor light absorption can be omitted by a complex formation in which the organic ligand plays the role of an “antenna”. Such a ligand effectively transfers the absorbed energy to the europium ions, improving the f-f transitions and thus increasing the red luminescence [2,39,40,41,42,43]. The incorporation of a lanthanide complex as admixtures in polymeric matrices during the synthesis routes mentioned above gives materials in block forms [30,32,44] while hybrid materials in film forms are often formed as the result of solvent evaporation from the mixture of dissolved solid metal complex and matrix [45].

Herein, we present investigations aimed at the two-stage in situ synthesis and characterization of BPA.DA-NVP@Eu_2_L_3_ hybrid materials on the grounds of the bisphenol A glycerolate diacrylate and N-vinylpyrrolidone monomers as the polymeric matrix and europium(III) quinoline-2,4-dicarboxylate complex (Eu_2_L_3_) as a luminescent dopant. This metal complex was chosen based on our previous studies on the structure and properties of the lanthanide coordination polymers constructed from a quinoline-2,4-dicarboxylate ligand which behaves as an effective coordinating agent and sensitizer of Eu(III) ions luminescence [45]. Two-stage synthesis includes the formation in situ europium(III) complex (Eu_2_L_3_) at concentrations of 0.1, 0.2, 0.5, 1, and 2% wt. and the main UV photopolymerization process leading to the final uniformly doped polymer materials. A different content of the additive was applied for the purpose of determining the effect of the admixture concentration on the luminescence, thermal, and mechanical properties of the materials which were investigated in detail. Additionally, a pure polymeric matrix as well as hybrid materials based on the precursors of the europium(III) complex, i.e., Eu(NO_3_)_3_ and quinoline-2,4-dicarboxylic acid (H_2_L), were obtained and tested. A comparison of the properties of these materials with the hybrid materials being the main subject of this study allowed us to better determine the impact of the Eu_2_L_3_ admixture on the structure and characteristics of the materials. The NMR and infrared spectroscopy were applied for the conformation of the formation of the hybrid materials and a better understanding of the impact of the dopant addition on the polymeric matrix features. The emission, excitation spectra, lifetime, and quantum yield measurements allowed for the determination of the luminescence properties of materials concerning the europium(III) complex content. Thermal stability and thermal decomposition mechanisms both in the oxidative and inert atmosphere were determined via the TG-DTG-DSC and TG-FTIR methods. We have also focused on the estimation of the mechanical properties of hybrid materials functionalized by the europium(III) complex dopant. To the best of our knowledge, these investigations are rather omitted [46] for such types of hybrid materials while they are routinely conducted for pure polymeric matrices [47,48,49]. Awareness of the mechanical properties of the tested materials will allow for the design of materials with optimal compositions, in which the functional dopant will bring the expected properties (in our case luminescence) [20,42,43,50], without a significant deterioration of the matrix properties or obtaining new features, beneficial in the context of their target application.

## 2. Results and Discussion

**Scheme 1:**
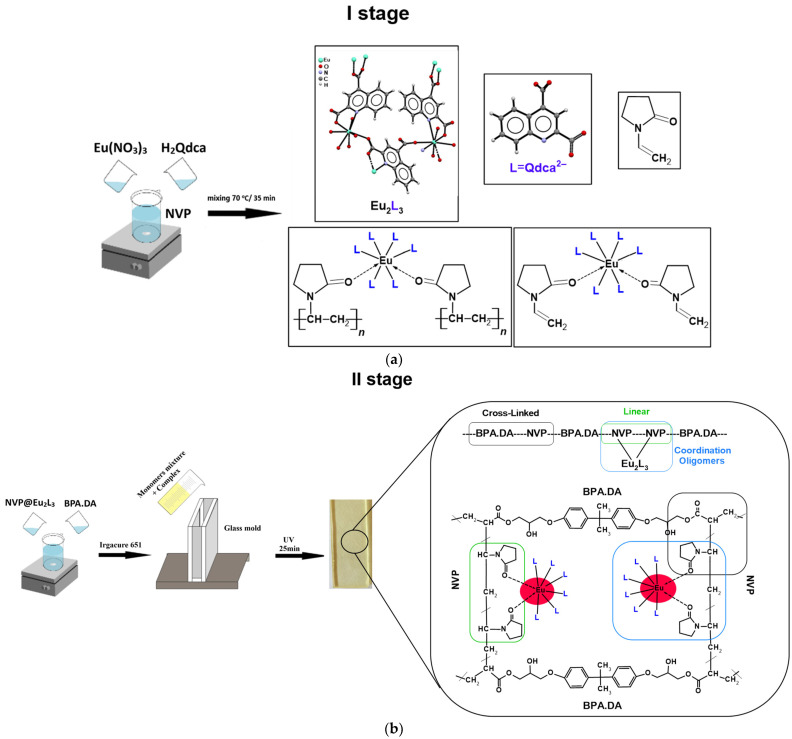
Schemes of the synthesis of hybrid materials with proposed structures of products at (**a**) the first stage of the synthesis of materials with possible formed coordination moieties; (**b**) the second stage of the synthesis of hybrid materials.

**Figure 1:**
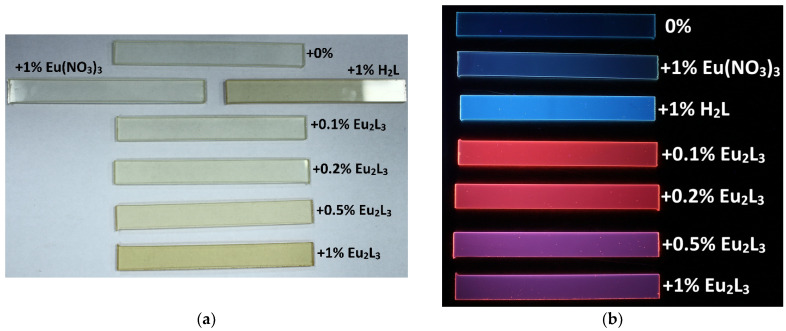
Images of hybrid materials with different contents of the europium(III) complex and substrates used in DMA analysis: (**a**) in daylight; (**b**) at UV light (364 nm).

A series of homogeneous hybrid materials were synthesized based on a cross-linked BPA.DA-NVP matrix and europium(III) complex as a luminescent dopant during the UV polymerization process (Figure 1). The in situ approach was utilized to form a luminescent dopant evenly distributed throughout the volume of the hybrid materials. This route allowed for overcoming the problem connected with the solubility of pre-synthesized powder europium(III) quinoline-2,4-dicarboxylate [51] in precursors of the polymeric matrix. Various contents (in stoichiometric ratio 2:3) of metal complex substrates (Table S1), i.e., europium(III) nitrate(V) and quinoline-2,4-dicarboxylic acid (H_2_L), were dissolved in N-vinylpyrrolidone to form the in situ coordination dopant (Scheme 1a). Taking into account the fact that not only the incorporated quinoline-2,4-dicarboxylate ligand (as a result of H_2_L deprotonation in alkaline NVP solution) will coordinate Eu(III) ions, but also NVP and/or PVP moieties may coordinate metal ions through carbonyl oxygen atoms [52], the formation of different coordination assemblies was assumed (Scheme 1a). Because we are unable to determine the exact composition of the resulting europium(III) complexes, further in the work we will use the abbreviation Eu_2_L_3_ to define the dopant. In the second stage of the synthesis of the materials, the BPA.DA monomer was mixed with NVP-Eu_2_L_3_ and the UV polymerization took place, yielding the hybrid BPA.DA-NVP@Eu_2_L_3_ materials with a 0.1%, 0.2%, 0.5%, 1%, and 2% concentration of the dopant (Figure 1). For comparative purposes, materials based on the BPA.DA-NVP matrix were obtained, with the addition of individual substrates, 1% wt. of H_2_L and 1% wt. of Eu(NO_3_)_3_.

The obtained hybrid materials are characterized by high transparency with a shade of yellow color. Hybrid materials take on a more intense yellow color and are slightly turbid with an increasing concentration of the lanthanide(III) complex (Figure 1a). A comparison of all materials including the free matrix, separate components of the metal dopant as well as those containing different contents of the europium(III) complex allow us to conclude that quinoline-2,4-dicarboxylic acid (or its anion) is responsible for the intensity of the yellow color in the tested samples. It is also worth mentioning that the elasticity of the obtained materials increases with the growing content of the admixture. Material with a 2% content of the dopant is the most elastic.

### 2.1. NMR Analysis

**Figure 2:**
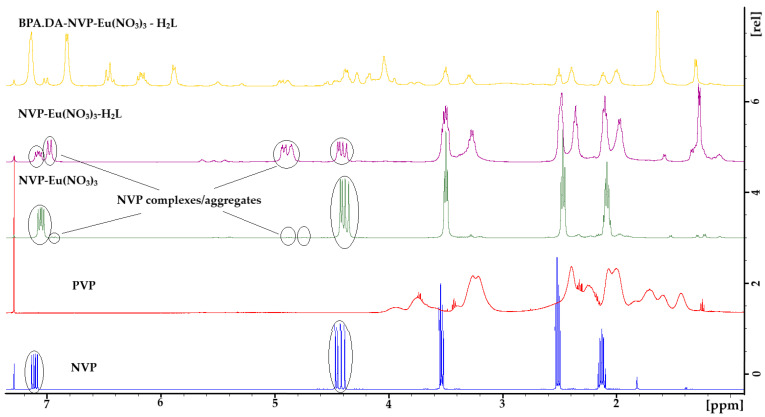
A combined ^1^H NMR spectra of NVP (blue), PVP (red), NVP with dissolved Eu(NO_3_)_3_ (green), NVP with dissolved Eu(NO_3_)_3_ and ligand (violet), a mixture of NVP, BPA, Eu(NO_3_)_3,_ and ligand (yellow).

The course of the formation of the hybrid material has been monitored using the NMR technique. In Figure 2, the combined ^1^H NMR spectra of several samples has been presented. All samples were prepared using chloroform-d as a solvent and in each case, a good solubility suitable for NMR analysis has been achieved.

**Figure 3:**
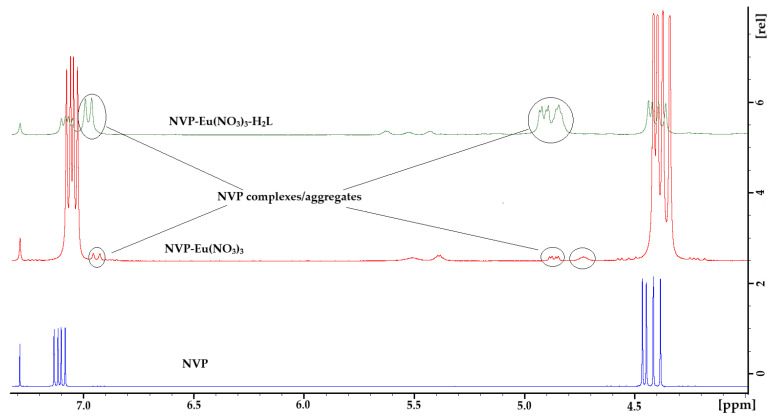
A combined inset of ^1^H NMR spectra of NVP (blue), NVP with dissolved Eu(NO_3_)_3_ (red), and NVP with dissolved Eu(NO_3_)_3_ and ligand (green).

The most characteristic signals in N-vinylpyrrolidone (NVP) (blue line), appearing as doublets or a doublet of doublets at 4.40 ppm, 4.46 ppm, and 7.11 ppm, respectively, belong to vinyl moiety bonded to a nitrogen atom. Polymerization of NVP yields polyvinylpyrrolidone (PVP) which lacks signals of this substituent in the ^1^H NMR spectra (red line). The mixing of europium(III) nitrate(V) with NVP resulted in a complete dissolution of the salt after a short heating of the mixture at 70 °C. The NMR analysis of this mixture initially showed no additional new signals (spectra not shown) but after heating for 3 h at 70 °C, the ^1^H NMR analysis revealed the presence of three new signals in the vinylic region, at 4.69–4.78 ppm, 4.83–4.90 ppm, and 6.94 ppm, respectively (Figure 3). The relative intensity of these three signals (1:1:1) points toward the presence of another compound possessing vinylic moiety. Based on the multiplicity, it can be concluded that there is a strong shielding of the signal of the starting NVP at 7.11 ppm which appears at 4.83–4.90 ppm in the newly formed species and a remarkable deshielding of the signal at 4.40 ppm in the starting NVP which appears at 6.94 ppm in the observed species. The third signal of the vinylic hydrogen appearing at 4.46 ppm in the starting NVP has been observed at 4.69–4.78 ppm, suggesting a small deshielding of the signal. All this also suggests the formation of a complex between the NVP and europium(III) ions and a remarkable change in the electron density around the *N*-vinylamido group. The nature of this complex cannot be judged based on the NMR analysis but based on the literature data, it can be assumed that the coordination occurs through carbonyl oxygen as the more appropriate ligating center of europium [53]. Another interesting feature of the newly formed species derived from NVP was associated with the multiplicity of the analyzed signals. In the starting NVP, the signal at 7.11 ppm appears as a doublet of doublets due to the different coupling constants of a vinylic proton with two protons of a vinylic CH_2_ group with the coupling constants being *J*_H-H_ = 9.1 Hz for *cis* hydrogen and *J*_H-H_ = 15.9 Hz for *trans* hydrogen, respectively. In the newly formed species, this signal appears as a doublet of doublets with *J*_H-H_ = 5.4 Hz for *cis* hydrogen and *J*_H-H_ = 14.4 Hz for *trans* hydrogen, respectively. Decreasing coupling constant values are usually a consequence of the complex formation between a ligand and a metal center.

When both europium(III) salt and a ligand were dissolved in warm NVP (Figure 2, pink line), the intensity of new signals at 4.69–4.78 ppm, 4.83–4.90 ppm, and 6.94 ppm increased remarkably compared to the mixture containing the sole europium salt. This might suggest the formation not only of a NVP-Eu_2_L_3_ complex but rather the formation of aggregates containing NVP, europium(III) ions, and a ligand. This could include the formation of relatively stable aggregates via hydrogen bonding between a ligand and NVP but also between two or more NVP molecules.

One should also mention that the formation of PVP via Lewis acid-catalyzed polymerization of NVP has not been detected using the NMR technique although this cannot be completely excluded due to the low sensitivity of the NMR analysis and overlapping of the signals.

### 2.2. ATR/FTIR Analysis

**Figure 4:**
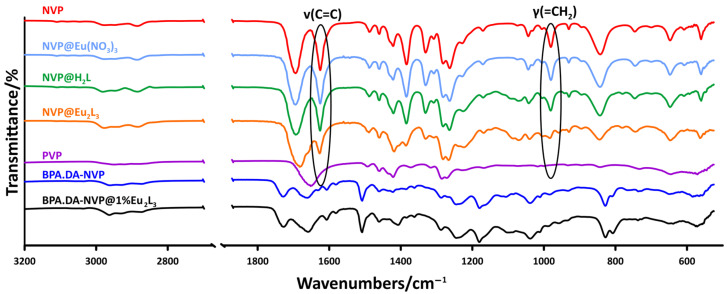
Comparison of the ATR/FTIR spectra of products formed in the first and the second stage of the synthesis of hybrid materials with infrared spectra of dissolved in NVP substrates of europium(III) complex, NVP, and PVP.

The comparison of the infrared spectra of the reaction mixture obtained in the first stage of the synthesis (NVP-Eu_2_L_3_) with the spectra of the pure NVP monomer and poly-vinylpyrrolidone (PVP) suggests that there is a partial linear polymerization of N-vinylpyrrolidone (NVP) to poly-vinylpyrrolidone (PVP) (Figure 4). This is confirmed by the decrease in the intensity of the bands at wavenumbers 1624 and 980 cm^−1^ originating from the stretching v(C=C) and out-of-plane deformation γ(=CH_2_) vibrations, respectively, as the result of polymer formation [54]. The polymerization of the NVP monomer is initialized most probably by the formation of europium(III) complexes as can be proposed based on the lack of changes in the intensity of those bands in the FTIR spectra of the NVP-H_2_L and NVP-Eu(NO_3_)_3_ materials. The reaction mixture obtained in the first stage of the synthesis is still characterized by the content of free NVP monomers, which later take part in cross-linking with BPA.DA, and also contains NVP-Eu_2_L_3_ coordination oligomers. The infrared spectra of free NVP, NVP@Eu(NO_3_)_3,_ and NVP@H_2_L are dominated by a strong band at 1694 cm^−1^ derived from the stretching vibrations of a carbonyl group v(C=O) characteristic of a five-membered lactam ring. This band is shifted to lower wavenumber 1690 cm^−1^ in the spectrum of the NVP@Eu_2_L_3,_ most probably as the result of covalent bond formation between the oxygen atom of the carbonyl group and Eu(III) ion. In the spectrum of PVP, this band appears at 1651 cm^−1^. The ATR/FTIR spectra of all samples based on NVP exhibit vibrations at 2976 and 2884 cm^−1^ from the asymmetric and symmetric stretching vibrations of methylene groups v(CH_2_) as well as bending modes δ(CH_2_) of such groups at 1421 and 1372 cm^−1^. The band at 1285 cm^−1^ was ascribed to the stretching ν(C-N) vibrations. Quite a strong band at 843 cm^−1^ was associated with the bending δ(=C-H) vibrations [55].

The infrared spectra of the matrix (BPA-NVP) as well as hybrid materials are given in Figure 4, Figures S1 and S2. All spectra are dominated by the bands derived from the bisphenol A glycerolate diacrylate (BPA.DA) compound. The band from the stretching vibrations of the hydroxyl groups ν(OH) from BPA.DA is observed in the range of 3100–3660 cm^−1^. In the spectrum of BPA.DA-NVP, the bands derived from the asymmetrical stretching ν_as_(CH_2_) and symmetrical stretching ν_s_(CH_2_) vibrations are observed at 2961 and 2926 cm^−1^. The intense bands at 1727 and 1660 cm^−1^ were assigned to the stretching vibrations of the carbonyl groups C=O from the N-vinylpyrrolidone and ester moieties. The bands recorded at 1630, 1606, and 1507 cm^−1^ were assigned to the stretching vibrations ν(C_Ar_C_Ar_) and ν(CN) of aromatic and pyrrolidone rings. The combination of bands at 1460, 1420, and 1385 cm^−1^ were assigned to the δ_as_(CH_3_), δ_s_(CH_2_), and δ_s_(CH_2_), respectively. In the region 1300–1000 cm^−1^, the bands’ characteristic of the stretching vibrations ν(C–O) group of esters from the BPA.DA moieties are observed. The infrared spectra of all obtained hybrid materials exhibit several bands at 1287, 1244, 1179, 1103, and 1036 cm^−1^ which can be ascribed to the stretching vibrations of C–(C=O)–C and C–O–C moieties [46]. The out-of-plane deformation γ(C_Ar_H) mode of benzene rings appears at 827 cm^−1^. The bands derived from the ligands of doped complexes are almost invisible in the spectra of the obtained materials due to their low concentrations. Moreover, the bands from the complexes overlapped those from the polymeric matrix.

### 2.3. Luminescence Properties

The photophysical properties of the hybrid BPA.DA-NVP@Eu_2_L_3_ materials doped with different contents of the europium(III) complex with quinoline-2,4-dicarboxylate ligand were studied using the emission, excitation spectra, lifetime, and quantum yield measurements.

**Figure 5:**
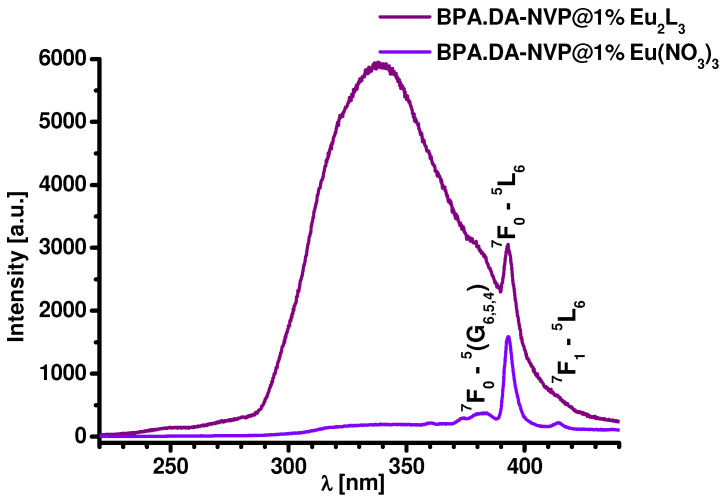
Excitation spectra of BPA.DA-NVP@1%Eu_2_L_3_ and BPA.DA-NVP@1%Eu(NO_3_)_3_ materials, λ_ob_ = 618 nm.

The excitation spectra of the BPA.DA-NVP@1%Eu_2_L_3_ and BPA.DA-NVP@1%Eu(NO_3_)_3_ are shown in Figure 5. In the case of the BPA.DA-NVP@1%Eu_2_L_3_ material, a weak, sharp band at 393 nm and one intense broad band, attributed to ligand-centered π–π* transitions, with a maximum of 340 nm are present. The spectrum of the BPA.DA-NVP@1%Eu(NO_3_)_3_ consists of three weak bands corresponding to the *f*-*f* transitions in the europium(III) ion. The use of the mentioned excitation wavelength values for these samples provides the typical red luminescence lines of the Eu(III) ion at nearly 580, 595, 620, 650, and 700 nm, attributed to the ^5^D_0_-^7^F_J_ (J = 0–4) transitions, respectively [56].

**Figure 6:**
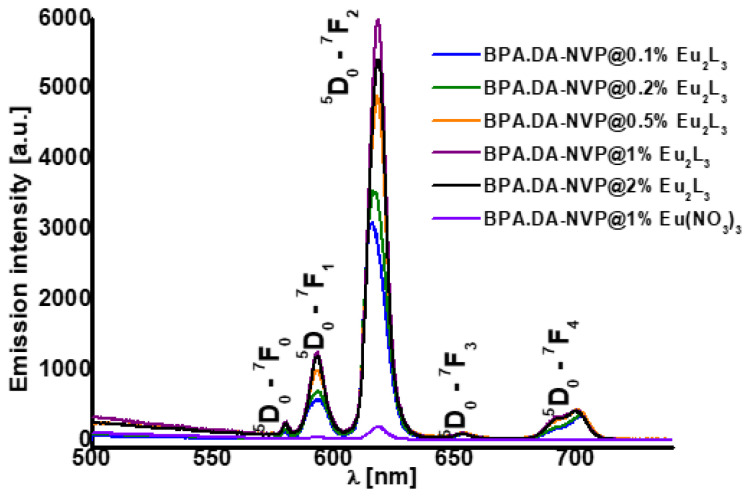
Comparison of the emission intensity of materials with Eu(III) complex and Eu(NO_3_)_3_ in BPA.DA-NVP matrix, λ_ex_ = 340 nm.

Figure 6 shows the luminescence spectra of the hybrid materials with different concentrations (0.1%, 0.2%, 0.5%, 1%, and 2% wt.) of the Eu_2_L_3_ complex and for comparison, the BPA.DA-NVP matrix doped with Eu(NO_3_)_3_ excited with λ = 340 nm, while Figure S3 shows the emission spectra of the pure matrix and the matrix doped with the H_2_Qdca (H_2_L) ligand under the same experimental conditions. The emission spectra present the two most intense bands at 594 and 618 nm which are associated with the ^5^D_0_-^7^F_1_ and ^5^D_0_-^7^F_2_ (exhibiting hypersensitivity) transitions. In addition, luminescence from the matrix was observed in the hybrid material, slightly affecting the character of the emission spectra. All materials with the Eu(III) complex display a higher emission intensity in comparison with the Eu(NO_3_)_3_ salt. The observed increase in the Eu(III) luminescence intensity is a result of an energy transfer from the ligand L^2-^ to the metal ion as the complex formation effect. As can be seen from this figure, material containing 1% of the Eu(III) complex shows the most intense luminescence. The material with the higher concentration of the dopant (2% wt.) exhibits a lower emission intensity in comparison to the BPA-DA-NVP@1%Eu_2_L_3_ due to the effect of concentration quenching due to the formation of aggregates [2]. For the BPA.DA-NVP@H_2_L material, a weak luminescence of the free H_2_L ligand was observed in the region of the europium ion emission (Figure S3). When the samples were excited with a wavelength of 393 nm, the highest emission intensity was also observed for a sample containing 1% of the europium(III) complex (Figure S4).

Table 1 collects the luminescence lifetime results and quantum yields measured for the BPA-DA-NVP@Eu_2_L_3_ materials with different concentrations of the europium(III) complexes as well as the ratio of the emission band intensity. The lifetime values were calculated using the bi-exponential decay method (Figure S5) with Equation (1):

(1) $$I=A_{1}\times exp\left(\frac{-x}{\tau_{1}}\right)+A_{2}\times exp\left(\frac{-x}{\tau_{2}}\right)$$

where *I* is the luminescence intensity at time *x*, A is the amplitude, and *τ* is the emission lifetime.

The calculated luminescent lifetimes increase, as in the case of the emission intensity, with the increase in the percentage of the europium(III) complex in the matrix, reaching the maximum value for the system containing 1% of the complex. The results of the band ratio show that in the analyzed systems, there is a systematic, slight increase in the symmetry around the Eu(III) ion.

For the full luminescence characteristics, the emission quantum efficiency was determined for all samples containing the europium(III) dopant. The emission quantum efficiency of the ^5^D_0_ emitting level of the Eu(III) ion was determined according to Equation (2) [57]:

(2) $$\phi =\frac{I_{em\;Eu^{3+}}}{I_{\mathrm{ex}\;La_{2}O_{3}}-I_{ex\;Eu^{3+}\;}}\cdot 100\%$$

where *ϕ* is the quantum yield, *I_emEu3+_* is the integrated intensity of the sample luminescence, and *I_ex La2O3_* and *I_ex Eu3+_* are the integrated intensities of the scattered excitation radiation not absorbed by the reference (La_2_O_3_) and integrated intensities of the scattered excitation light for the investigated material, respectively. All procedures were carried out in accordance with [56,57]. The sample containing 0.1% of the Eu(III) complex reached the highest *ϕ* values among these materials. The determined-by-us values of the absolute luminescent quantum yields (*ϕ*) of the ^5^D_0_ europium ion excited state, measured using an integrating sphere, are similar or lower than those of the silica–polymeric and mesoporous hybrids containing these ions [58,59,60,61].

The lack of correlation between the lifetime and emission quantum yields is probably due to specific interactions between the ligand and the matrix and/or Eu(III) ions with the matrix, which influence these parameters [62].

### 2.4. Thermal Analysis in Air Atmosphere

**Figure 7:**
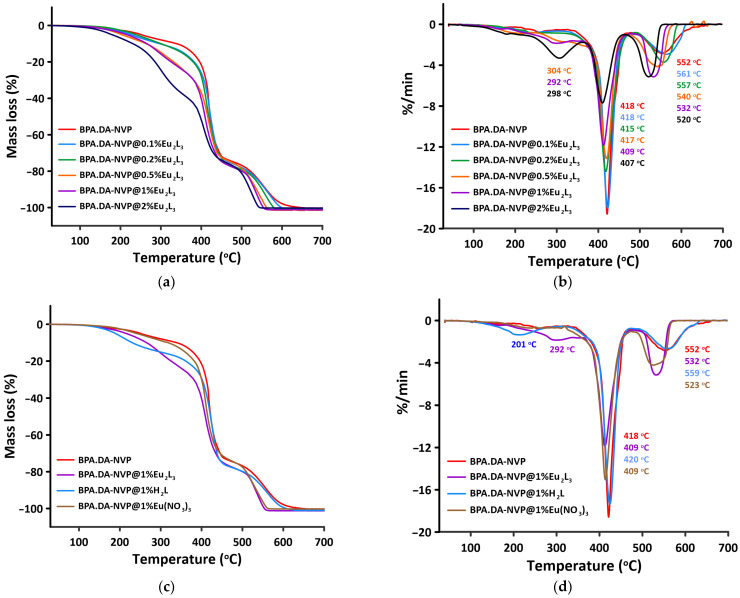
Comparison of TG and DTG curves of tested samples in air atmosphere: (**a**) TG and (**b**) DTG of the BPA.DA–NVP matrix and hybrid materials (BPA.DA-NVP@0.1/0.2/0.5/1/2%Eu_2_L_3_); (**c**) TG and (**d**) DTG of the BPA.DA-NVP matrix and materials doped with @1%Eu_2_L_3_/H_2_L/Eu(NO_3_)_3_. The temperatures of the maximum rates of mass losses (T_DTGmax_) along with DTG curves are given.

Taking into account the profiles of the TG and DTG curves (Figure 7), it can be a statement that the thermal decomposition of all investigated samples occurs in the main three overlapping stages (Table 2). Additionally, the increase in the additive content results in a more complex decomposition process. The first mass loss took place in the temperature range of 90–355 °C and is generally connected with continuous mass changes in the range of 8.7–37.8%. The greatest mass loss in this stage was observed for the BPA.DA-NVP@2%Eu_2_L_3_ material. The next step in the decomposition of the tested samples was recorded in the range of 256–474 °C accompanied by significant mass changes of 36.3–69.8%. The last stage of thermal degradation was observed in the range of 445–645 °C with a mass loss of 22.5–29.0%.

The profiles of the DTG curves along with the temperature of the maximum rate of mass loss (T_DTGmax_) in the main three steps confirm changes in the mechanisms of the thermal decomposition of the tested samples due to the effect of the dopant addition (Figure 7b,d).

The thermal behavior stability (Figure 7) of the studied materials was described in relation to the temperature of specific mass losses, given in Table 3. As can be suggested from the thermal data, the addition of Eu(NO_3_)_3_ increases the thermal stability of the material in comparison to the parent matrix, taking into account the temperature of the 1% mass loss. A similar effect was observed for the BPA.DA-NVP@0.2%Eu_2_L_3_ material. The incorporation of 1% wt. of H_2_L as well as 0.1, 0.5, 1, and 2% of Eu_2_L_3_ leads to the decrease in the thermal stability by 18, 7, 4, 7, and 32 °C, respectively. The greatest decrease in the temperature was observed for the highest amount of metal complex addition (2% wt. of Eu_2_L_3_). At a higher temperature, the investigated materials started to decompose. It is worth mentioning that the shapes of the TG–DTG curves of the BPA.DA-NVP, BPA.DA-NVP@0.1%Eu_2_L_3_, BPA.DA-NVP@0.2%Eu_2_L_3,_ and BPA.DA-NVP@1%Eu(NO_3_)_3_ materials up to about 450 °C are very similar. This observation points to the fact that the supplementation of the polymeric matrix by a very small amount of metal complex or inorganic salt does not change its thermal properties drastically. The temperature recorded at mass losses of 5, 20, and 50% for these materials is lower by 11–24, 12–17, and 1–7 °C in relation to the free matrix. For the BPA.DA-NVP@0.5%Eu_2_L_3_, BPA.DA-NVP@1%Eu_2_L_3_, BPA.DA-NVP@2%Eu_2_L_3,_ and BPA.DA-NVP@1%H_2_L materials, the temperature changes in the above mentioned mass losses regarding to the matrix are 35–72, 35–111, and 1–25 °C, respectively. The materials containing 0.5, 1, and 2% wt. of the europium(III) complex as well as 1% wt. of H_2_L show an additional mass loss in the temperature range of 200–400 °C.

The shapes of the DTG curves (Figure 7b,d) allow for concluding that the highest rate of mass loss in the first stage (I) of the decomposition of the tested materials is observed for the material doped with 2% wt. of Eu_2_L_3._ In the second stage (II) of the decomposition of the materials, the highest rate and T_DTGmax_ were observed for the free matrix, BPA.DA-NVP@1%H_2_L, and BPA.DA-NVP@0.1%Eu_2_L_3_. The addition of Eu(NO_3_)_3_ and increase in the additive contents (above 0.1% wt. of Eu_2_L_3_) lead to the decrease in the temperature of the maximum rate of mass loss while the incorporation of H_2_L very slightly increases such a temperature. The highest rate and the lowest T_DTGmax_ in the third stage of the decomposition of the investigated materials were noticed for the BPA.DA-NVP@1%Eu_2_L_3_ and BPA.DA-NVP@2%Eu_2_L_3_. On the other hand, the lowest rate of mass loss and highest T_DTGmax_ in this stage of decomposition were noticed for the free matrix, BPA.DA-NVP@1%H_2_L, BPA.DA-NVP@0.1%Eu_2_L_3,_ and BPA.DA-NVP@0.2%Eu_2_L_3_.

The DSC curves (Figures S6 and S7) recorded up to about 450 °C are dominated by overlapping and hardly distinguishable endothermic effects, most likely due to the melting of the polymeric matrix and cleavage of covalent bonds in polymeric frameworks and applied dopants. The thermal cracking inside the materials gives fragments of polymer molecules that burn at higher temperatures. All materials above 450 °C show the second distinct mass loss, which provides a total decomposition of the tested materials. The highest rate of mass change and the lowest temperature of T_DTGmax_ were observed for the materials with the highest content of Eu_2_L_3_ (1% wt. and 2%wt.) and Eu(NO_3_)_3_. The free matrix BPA.DA-NVP and BPA.DA-NVP@1%H_2_L materials decompose at the highest temperature. This last step of mass loss is accompanied by a strong exothermic effect visible on the DSC curves at a maximum of effect in the temperature range of 500–550 °C caused by the burning processes of organic moieties present in the solid residues formed in the earlier processes. Only traces of Eu_2_O_3_ as the solid products of the materials’ decomposition were observed for materials with 1 and 2% wt of the complex.

### 2.5. Thermal Analysis in Nitrogen

**Figure 8:**
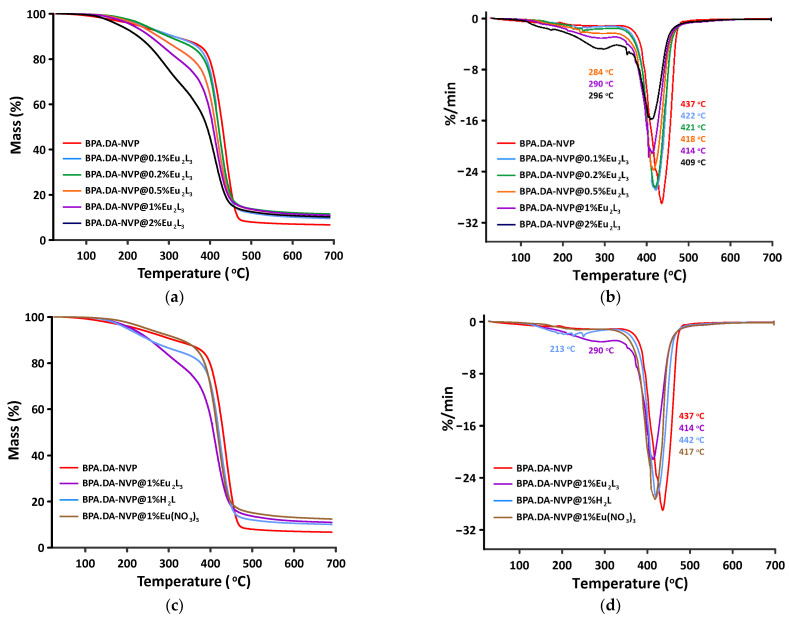
Comparison of TG and DTG curves of tested samples in nitrogen atmosphere: (**a**) TG and (**b**) DTG of the BPA.DA–NVP matrix and hybrid materials (BPA.DA-NVP@0.1/0.2/0.5/1/2%Eu_2_L_3_); (**c**) TG and (**d**) DTG of the BPA.DA-NVP matrix and materials doped with @1%Eu_2_L_3_/H_2_L/Eu(NO_3_)_3_. The temperatures of the maximum rates of mass losses (T_DTGmax_) along with DTG curves are given.

The thermal behavior of the tested samples was also investigated in the nitrogen atmosphere (Table 3, Figure 8). Regarding the temperature at the 1% mass loss for the TG curves, the increase in thermal stability in the nitrogen atmosphere in comparison to the oxidative atmosphere is observed for all hybrid materials. Similarly, as in the air, the BPA.DA-NVP@0.2%Eu_2_L_3_ and BPA.DA-NVP@1%Eu(NO_3_)_3_ materials are characterized by the highest stability. It is noteworthy to mention that the free matrix shows the lowest temperature at a 1% mass loss. Further heating results in gradual mass losses of the materials in two main stages. The profiles of the TG curves and thermal data clearly show that the incorporation of greater amounts of dopants causes the decomposition process shifts toward a lower temperature. This trend is especially seen for mass losses higher than 5%. In general, the overall effect of the increased amount of additives on the stability is in agreement with those observed in the air atmosphere but this influence is more expectable. Above ca. 350 °C, the second stage of decomposition with a rapid mass loss up to ca. 500 °C can be distinguished (for hybrid materials at lower temperatures). In this stage, the free matrix shows the highest rate of mass loss as well as T_DTGmax_ temperature. The increase in the metal complex content in the hybrid materials results in the decrease in the T_DTGmax_ temperature. Further heating causes only very small mass changes and leads to the formation of solid residues (6.7–13.2%), i.e., unburnt carbon and europium(III) oxide (for materials containing europium compound).

**Figure 9:**
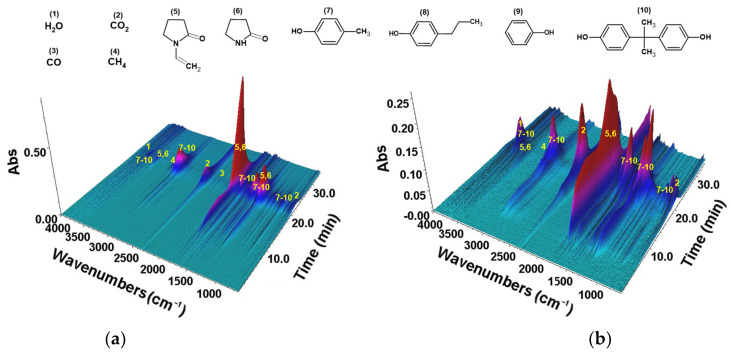
The 3D projection of FTIR spectra of volatile product decomposition of (**a**) free matrix (BPA.DA-NVP), (**b**) hybrid materials (BPA.DA-NVP@2%Eu_2_L_3_) in the nitrogen atmosphere. Evolved gases: **1**—H_2_O; **2**—CO_2_; **3**—CO; **4**—CH_4_; **5**—N-vinylpyrrolidone (NVP); **6**—2-pyrrolidone; **7–10**—4-methylphenol, 4-propylphenol, phenol, Bisphenol A.

The pyrolysis process of the BPA.DA-NVP and BPA.DA-NVP@2%Eu_2_L_3_ was also monitored by recording the FTIR spectra of the gaseous products of their decomposition in nitrogen (Figure 9). The stacked infrared spectra of the evolved products of the material degradation show that molecules of water, carbon dioxide, and pyrrolidone derivatives are evolved at first (Figure S8). Decomposition of the matrix leads to the release of such gases after 5 min of heating (127 °C) while for the hybrid material, after 4 min (110 °C). The weak bands of stretching and deformation vibrations of the water molecules are observed in the ranges of 4000–3500 and 1800–1300 cm^−1^, respectively. Carbon dioxide molecules give bands in the ranges of 2358–2310 and 700–600 cm^−1^ (the strongest at 668 cm^−1^) due to the stretching and deformation vibration modes. One of the main products of material degradation is 2-pyrrolidone as the product of the polymeric matrix degradation. The most intense band appears at 1750 cm^−1^ due to the stretching vibrations of the carbonyl group from the lactam ring. The bands at 2969, 2884 cm^−1^, and those at 1386 and 1269 cm^−1^, are ascribed to the stretching and deformation vibrations of the aliphatic C-H groups and the stretching vibrations of the C-N group. The confirmation of 2-pyrrolidone formation is also a band at 3481 cm^−1^ due to the presence of N-H stretching vibrations [46,63].

On the other hand, it can also be concluded that the N-vinylpyrrolidone monomer also evolved during the heating of the materials as the product of depolymerization. The FTIR spectra exhibit medium strong bands at 1635 and 841 cm^−1^ characteristic for the stretching vibrations of C=C and deformation vibrations of the =C-H groups, respectively, in alkenes that may be indicative of vinyl group appearance [64]. The abovementioned products dominated the first stage of the decomposition of the BPA.DA-NVP and BPA.DA-NVP@2%Eu_2_L_3_ materials and are evolved up to about 385 °C (18 min) and 215 °C (9.40 min), respectively.

In the next steps, the further decomposition of the polymeric materials takes place. In addition to the pyrrolidone/N-vinylpyrrolidone-evolved compounds, the FTIR spectra point out the release of other volatile products which perfectly fit with the reference spectra (Figures S9–S12) of several different compounds such as 4-methylphenol, 2-methylphenol, bisphenol, phenol, methane, and carbon monoxide.

The presence of phenol derivatives is reflected in the most diagnostic and strongest bands at 1255 and 1176 cm^−1^ derived from the stretching vibrations of the C_Ar_-O groups [65]. Absorption bands additionally appear due to the stretching vibrations of OH groups at 3648 cm^−1^, stretching vibrations of C_Ar_C_Ar_ at 1602 and 1507 cm^−1^ as well as out-of-plane deformation vibrations of C_Ar_H at 827 and 746 cm^−1^ from the aromatic ring [46]. Moreover, at a higher wavenumber range of 3100–3000 cm^−1^, several bands can be distinguished at 3085, 3067, and 3031 cm^−1^ assigned to the stretching vibrations of the C_Ar_-H groups. Along with phenols, methane molecules are evolved because of the diagnostic sharp band at 3015 cm^−1^ derived from the stretching vibrations of the C-H bonds. In addition to the carbon dioxide molecules which are evolved with a greater intensity in this stage, weak bands at 2150 and 2090 cm^−1^ in the infrared spectra are also seen due to carbon monoxide evolution [63]. It should be noted that the release of phenol derivatives occurs at a much lower temperature and with a greater intensity during the heating of the hybrid material compared to the matrix.

The most intense gas evolution during the degradation of the investigated materials takes place in the range of 20–25 min (426–527 °C). The single FTIR spectra recorded at 460 °C both for the matrix and BPA.DA-NVP@2%Eu_2_L_3_ material clearly show a different quantitative composition of evolved gases as can be suggested by the ratio of the intensity of bands C=O/C_Ar_-O. For the free matrix, this ratio is about 3 which indicates the domination of pyrrolidone moieties while for the hybrid material, this ratio is about 1, pointing out that phenol derivatives are the second main components of the evolved gaseous mixture. Above 500 °C, only traces of gaseous products of the degradation of the tested materials (mainly carbon oxides and water) are evolved.

### 2.6. DMA Analysis

**Figure 10:**
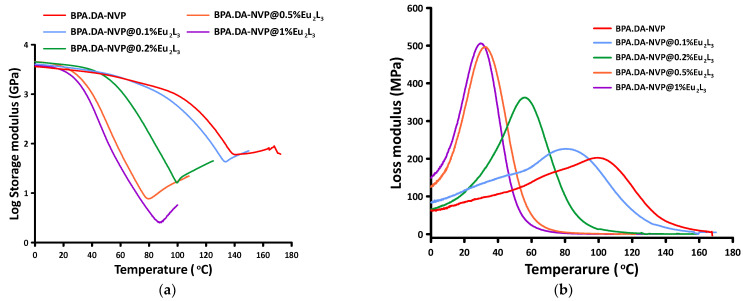

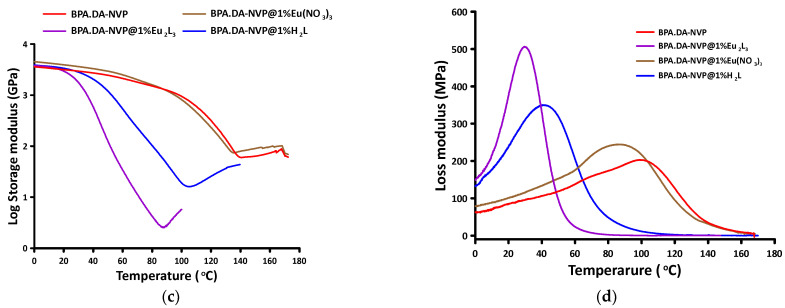
Storage (E′) and loss (E″) modulus versus temperature (**a**,**b**) polymer matrix (BPA.DA-NVP) and hybrid materials doped with europium(III) complex; (**c**,**d**) comparison of the polymer matrix with hybrid materials obtained with the addition of only 1% of substrates (Eu(NO_3_)_3_ and H_2_L, as well as doped with 1% of europium(III) complex.

**Figure 11:**
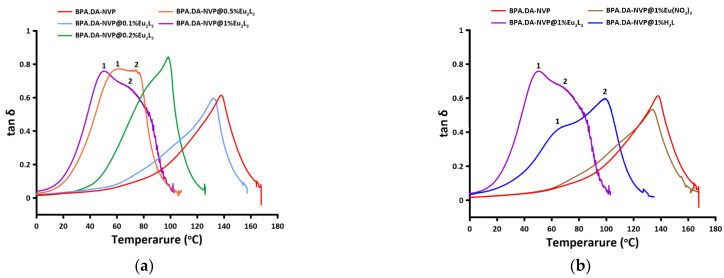
Tangent δ (tan δ) versus temperature: (**a**) polymer matrix (BPA.DA-NVP) and hybrid materials doped with europium(III) complex; (**b**) comparison of the polymer matrix with hybrid materials obtained with the addition of only 1% of substrates (Eu(NO_3_)_3_ and H_2_L, as well as 1%-doped europium(III) complex; ^1,2^—two maxima of the tan δ_max_.

To study the effect of the different wt.% content of the europium(III) complex on the viscoelastic properties, a dynamic mechanical thermal analysis (DMA) was performed. The results from the DMA analysis of the tested samples are presented in Figure 10 and Figure 11 and Table 4.

Determining the glass transition temperature (T_g_) via three different methods, δ_max_, E′_onset,_ and E′′_max,_ generally shows a trend of the glass transition temperature of the obtained materials decreasing with the increasing content of the europium(III) complex or independent substrates (Table 4, Figure 10 and Figure 11).

By analyzing the shape of the storage modulus versus the temperature curves, it was observed that the storage modulus (E′) gradually decreases above 20 °C for the obtained materials (Figure 10a,c). The fastest change in the decay rate of E’ is observed for materials with the highest addition of the europium(III) complex (BPA.DA-NVP@0.5%Eu_2_L_3_, BPA.DA-NVP@1%Eu_2_L_3_) and ligand (BPA.DA-NVP@1%H_2_L). These changes also reflect the lowest glass transition temperatures (31.45 °C, 28.6 °C, and 38.88 °C, respectively) compared to the polymer matrix (BPA.DA), where T_g_ is 99.87 °C. From the shapes of the storage modulus and DSC curves (Figures S6 and S7), the area responsible for the partial cross-linking of the obtained materials is also determined. In the DSC curves, this area is observed for the range of 190–200 °C. A drastic reduction in the storage modulus (E’) is noticed in the region related to the glass transition temperature (T_g_) of the investigated materials. In this region, the chain segments, in contrast to the elastic energy range, are subject to increased displacement or rotational movements. This region is often referred to as the polymer softening region [66].

Determining the glass transition temperature as the position of the maximum value of the tan δ_max_ (°C) peak, a tendency to decrease the value of the glass transition temperature (tan δ_max_ (°C)) with the increasing addition of the europium(III) complex in comparison to the matrix polymer matrix (BPA.DA-NVP) is also observed. The only material with similar values of tan δ_max_ (°C) to the BPA.DA-NVP matrix (tan δ = 137.6 °C) is the BPA.DA-NVP@1%Eu(NO_3_)_3_ material (tan δ_max_ = 134.05 °C) (Figure 11, Table 4). The shape of the tan δ peaks and the full width at half maximum (FWHM) of the tan δ peak are used to measure the polymer heterogeneity. Based on the FWHM(°C) value in the series of the obtained materials, it can be concluded that the pure BPA.DA-NVP matrix is the most homogeneous (FWHM = 34.28 °C), while with an increase in the addition of the europium(III) complex, the heterogeneity of the materials increases. The highest FWHM values are observed for the BPA.DA-NVP@1%Eu_2_L_3_ and BPA.DA-NVP@1%H_2_L materials, which are 52.09 °C and 56.3 °C, respectively (Table 4). These materials show the greatest degree of heterogeneity. Also, the confirmation of the increased heterogeneity of the material is the presence of two maxima tan δ in the curves observed for the BPA.DA-NVP@0.5%Eu_2_L_3_, BPA.DA-NVP@1%Eu_2_L_3_, and BPA.DA-NVP@1%H_2_L materials, which may be the result of weaker interfacial interactions (hydrogen bonds and Van der Waals forces) between the polymer matrix and the formed europium(III) complex [67].

### 2.7. Flexural Test

The crack resistance was designated during the three-point bending determining the modulus of the flexural strength of the conventional yield strength and bending deformation. The test was carried out on samples (2 mm × 10 mm × 80 mm) of a pure polymer matrix and hybrid materials. The results of the flexural tests are presented in Table 5. The value of the Young’s modulus at the beginning with the increase content of the europium(III) complex increased to 3.79 and 3.90 GPa for the BPA.DA-NVP@0.1%Eu_2_L_3_ and BPA.DA-NVP@0.2%Eu_2_L_3_ materials (respectively), but no significant changes in the stresses and bending deflections were observed. Then, with the increase in the amount of the Eu_2_L_3_ complex in the materials, the Young’s modulus decreases to 2.98 GPa, 2.48 GPa, and 0.34 GPa for the BPA.DA-NVP@0.5%Eu_2_L_3_, BPA.DA-NVP@1%Eu_2_L_3,_ and BPA.DA-NVP@1%H_2_L samples, respectively. Also, for these samples, there are changes in the stress values toward smaller values of 75.77, 14.55, and 61.31 MPa (respectively), while the degree of deflection increases (Table 5). Reducing the stresses in the material causes a loss of stiffness, while increasing its flexibility. For the BPA.DA-NVP@1%Eu_2_L_3_ sample, the degree of deflection exceeds >7%, which also shows that the sample is the most flexible in the series. The observed changes may be the result of a weakening of the interactions between the polymer chains and the introduced europium complex, and an increase in the heterogeneity of the material with a higher content of the additive, which was previously confirmed by the DMA analysis.

### 2.8. Hardness Measurements

The measurements of hardness consisted of the vertical immersion of the indenter into the composite surface. The data of these parameters are expressed in the D scale in Table 6.

The hardness of the composites before and after the DMA analysis was in the range of 83–19 ShD. The hardness of the analyzed samples after the DMA analysis increased slightly by 2% compared to the samples before the DMA analysis, which is related to the cross-linking of the polymer matrix at higher temperatures. The highest hardness is observed for the sample (BPA.DA-NVP) without the functional additive; then, a decrease in the hardness was observed with the increase (wt.%) in the addition of the Eu_2_L_3_ complex. The lowest value was assigned to the sample with 2 wt.% of the content of the Eu_2_L_3_ complex. Additions of substrates Eu(NO_3_)_3_ and H_2_L also reduce the hardness of the material in a slight way (2–4%), but not as significantly as the addition of the whole complex. With an increase in the wt. % of the complex content in the hybrid material, its hardness decreases as follows: 0.1 wt.% →**0.85%**; 0.2 wt.% →**1.2%**; 0.5 wt.% →**27%**; 1 wt.% →**48.5%**; 2 wt.% →**75%**. These results show the increasing ductility of the polymeric hybrid materials based on the BPA.DA-NVP and the use of europium(III) complexes as the functional dopant which also have characteristics of the plasticizer properties.

## 3. Materials and Methods

Bisphenol A glycerolate (1 glycerol/phenol) diacrylate (BPA.DA), N-vinylpyrrolidone (NVP), and 2,2-dimethoxy-2-phenylacetophenone (Irgacure 651, IQ) were purchased from Sigma-Aldrich (Darmstadt, Germany). All the chemical reagents and materials were obtained from commercial sources and used without further purification. Hydrate of Europium(III) nitrate(V) (99.9%) was purchased from Merck (Darmstadt, Germany) and used without further purification. Quinoline-2,4-dicarboxylic acid (95%) was purchased from Merck (Darmstadt, Germany) and purified by heating with distilled water.

### 3.1. Synthesis of Hybrid Materials

NVP and BPA.DA monomers were used in proportions of 70:30% by weight. In the first stage, europium(III) nitrate(V) hydrate and quinoline-2,4-dicarboxylic acid were dissolved in the NVP monomer in stoichiometric amounts, forming together 0.1%, 0.2%, 0.5%, 1%, and 2% of the Eu_2_L_3_ complex in situ by mass throughout the hybrid material (Table S1). In the second stage, the mixture of NVP with the appropriate mass addition of the Eu_2_L_3_ complex was mixed with the BPA.DA monomer. Irgacure 651 was then added at 1% wt. as a photoinitiator. The prepared mixtures were heated at 80 °C to remove air bubbles. Well-homogenized mixtures were poured into glass molds (10 × 12 × 0.2 cm) with a Teflon spacer and exposed to UV radiation (8 lamps, each with a power of 40 W). The UV polymerization time was 25–30 min, and then, the resulting composites were heated at 80 °C for 2 h for final cross-linking. For comparative purposes, the same method was applied to obtain the pure matrix and materials containing admixtures with only 1% wt. of europium(III) nitrate(V) or quinoline-2,4-dicarboxylic acid. All materials were obtained in the form of blocks.

### 3.2. Instrumentation and Methods

The infrared spectra (ATR/FTIR) of the tested samples were recorded using a Nicolet 6700 spectrophotometer (Thermo Scientific, Waltham, US), equipped with the Smart iTR accessory (diamond crystal) in the range of 4000–600 cm^−1^.

The ^1^H NMR spectra (zg30 pulse program) were recorded with Bruker Ascend 500 MHz spectrometer in chloroform-d (Deutero, Kastellaun, Germany, 99.8 atom %D) as a solvent at room temperature. Chemical shifts are reported in ppm relative to the residual solvent peak. Samples for analysis were prepared as follows: 20 mg of the analyzed sample was placed in a 5 mm NMR tube, followed by adding chloroform-d (0.7 mL). The tube was closed by a stopcock and shaken intensely until a homogeneous solution was obtained.

Thermal analyses were carried out on a SETSYS 16/18 thermal analyzer (Setaram, Caluire, France) using the thermogravimetric (TG), derivative thermogravimetry (DTG), and differential scanning calorimetry (DSC) methods. The block samples (about 6–8 mg) were heated in the alumina crucibles up to 1000 °C at a heating rate of 10 °C min^−1^ in the dynamic air atmosphere (12.5 cm^3^ min^−1^). The thermograms (TG curves) along with the FTIR spectra of the volatile products of the decomposition were recorded by the Q5000 thermal analyzer (TA Instruments, TA Instruments, New Castle, DE, USA) coupled with the Nicolet 6700 spectrophotometer. The samples of 25–30 mg were heated in the dynamic nitrogen atmosphere (25 cm^3^ min^−1^) at a heating rate of 20 °C min^−1^ from room temperature to 700 °C in the open platinum crucibles. The gas cell of the spectrophotometer was heated up to 240 °C while the temperature of the transfer line was 250 °C.

The emission and excitation spectra in the UV–Vis range were recorded on a Hitachi F7000 Fluorescence Spectrometer using a 450 W Xenon lamp, with a wavelength range of 200–800 nm. Excitation and emission spectra were corrected for the instrumental response. The QuantaMasterTM 40 (Photon Technology International, Birmingham, UK) spectrophotometer equipped with an Opolette 355LD UVDM (Opotek Incorporation, Carlsbad, USA) tunable laser (excitation source), which had a repetition rate of 20 Hz as the excitation source and a Hamamatsu R928 photomultiplier as a detector, was used to measure luminescence decays. All measurements were collected at room temperature.

Dynamic mechanical analysis (DMA) was performed using DMA Q800 Analyzer TA Instruments (New Castle, DE, USA). Thermomechanical properties of the cured materials were determined from the storage modulus, loss modulus, and damping factor (tan δ_max_) versus temperature. Measurements for all samples were made in the scanning temperatures ranging from 0 to broken sample 190 °C, under natural air conditions, at a constant heating rate of 3 °C/min. The experiments were conducted using rectangular samples of dimensions close to 2 ± 0.1 mm thick, 10 ± 0.2 mm wide, and 35 ± 0.1 mm long. Mechanical properties were determined using a Zwick/Roell testing machine (model Z010, Zwick GmbH & Co. KG, Ulm, Germany). The specimen dimensions were 80 × 10 × 2 (±0.2) mm. The measurements were made at room temperature with a crosshead speed of 50 mm/min.

The hardness of samples was compared employing the Shore Hardness Tester Zwick/Roell Z010 (model Z010, Zwick GmbH & Co. KG, Ulm, Germany) in D scale. Five measurements were made for each sample and the average hardness was calculated for all samples.

## 4. Conclusions

In summary, we have successfully fabricated a series of novel luminescent, transparent, and homogenous hybrid materials based on the BPA.DA-NVP matrix endowed with different concentrations of europium(III) quinoline-2,4-dicarboxylate as a luminescent additive. Furthermore, investigations of the free polymeric matrix and materials doped with distinct precursors of additive were conducted to gain a deeper understanding of the mechanism of the formation of the final materials. The applied in situ strategy was very effective, rapid, and low energy-consuming and allowed for the formation of the effective luminescence dopant covalently bonded with the polymer matrix at the molecular level. Even a small amount of Eu_2_L_3_ (0.1% wt.) dopant could incorporate luminescence features into the material due to the proper choice of ligand in the europium(III) complex but also changed the mechanical properties. The most intense red luminescence was observed for the hybrid material with 1% wt. of the Eu(III) complex. The thermal stability and pathways of the degradation of the materials in air and nitrogen were studied in detail. It was shown that the thermal behavior of the tested samples strongly depends on the content and character of the used dopant. The gaseous products of the free matrix and hybrid material were identified. A higher dopant content in the hybrid materials caused increased elasticity while their hardness was reduced. Furthermore, the temperature of the glass transition decreased as the content of the admixture increased. These changes in the mechanical properties are highly informative from the processability perspective in the context of their potential application. In addition, this work showed that the rational molecular design can easily change the mechanical properties of the hybrid materials which may potentially be used in chemical sensing, security systems, and protective coatings against UV.

## Figures and Tables

**Table 1:** Spectral characteristics of Eu(III) ion in complex incorporated into BPA.DA-NVP matrix, (λ_ex_ = 340 nm).

Sample/%	0.1%	0.2%	0.5%	1%	2%
Lifetime τ/μs	*τ*_1_ = 78.1 ± 0.69 *τ*_2_ = 447.3 ± 0.84	*τ*_1_ = 93.42 ± 0.58 *τ*_2_ = 460.72 ± 0.78	*τ*_1_ = 119.29 ± 0.62 *τ*_2_ = 605.69 ± 1.56	*τ*_1_ = 143.57 ± 0.85 *τ*_2_ = 670.30 ± 1.95	*τ*_1_ = 147.71 ± 1.06 *τ*_2_ = 535.87 ± 1.38
$\eta =\frac{I_{618}}{I_{594}}$	5.20	5.07	4.86	4.71	4.50
*ϕ*/%	14.0	11.8	11.8	9.0	9.3

**Table 2:** Thermal data for investigated materials’ decomposition in air atmosphere.

Materials	Stage I		Stage II		Stage III	
	Temperature Range (°C)	Mass Loss (%)	Temperature Range (°C)	Mass Loss (%)	Temperature Range (°C)	Mass Loss (%)
BPA.DA-NVP	105–323	8.7	323–458	63.9	458–645	26.8
BPA.DA-NVP@0.1%Eu_2_L_3_	117–321	10.9	321–459	62.2	459–610	27.1
BPA.DA-NVP@0.2%Eu_2_L_3_	144–262	6.3	262–472	69.8	472–585	24.1
BPA.DA-NVP@0.5%Eu_2_L_3_	122–256	8.8	256–445	62.2	445–563	29
BPA.DA-NVP@1%Eu_2_L_3_	115–345	23.2	345–474	54.1	474–553	22.4
BPA.DA-NVP@2%Eu_2_L_3_	90–355	37.8	355–453	36.2	453–545	25.8
BPA.DA-NVP@1%Eu(NO_3_)_3_	128–334	10.8	334–468	61.9	468–564	27.3
BPA.DA-NVP@1%H_2_L	115–310	14.9	310–467	61.4	467–627	23.7

**Table 3:** Comparison of thermal behavior of investigated materials in air/nitrogen atmosphere.

Mass Loss (%)	Temperature (°C) for Materials BPA.DA-NVP with Different Content of Metal Complex and Substrates in Air/Nitrogen Atmosphere							
	0% Eu_2_L_3_	0.1% Eu_2_L_3_	0.2% Eu_2_L_3_	0.5% Eu_2_L_3_	1% Eu_2_L_3_	2% Eu_2_L_3_	1% Eu(NO_3_)_3_	1% H_2_L
1	144/106	137/145	165/164	140/155	137/145	112/123	153/161	126/140
5	251/223	227/238	238/239	203/226	216/211	179/179	240/246	189/200
20	396/398	384/391	379/384	331/356	325/321	285/278	382/385	361/376
50	422/430	423/420	418/420	416/413	409/406	397/392	415/409	421/420

**Table 4:** Data from dynamic mechanical analysis (DMA) of obtained materials.

Name of Sample	E*’_onset_* (°C)	E*’_20_* (GPa)	E*’’_max_* (°C)	tan δ_max_ (°C)	tan δ_max_	FWHM (°C)
BPA.DA-NVP	99.87	3.18	99.79	137.6	0.548	34.28
BPA.DA-NVP@0.1%Eu_2_L_3_	89.36	3.52	80.55	132.21	0.515	35.28
BPA.DA-NVP@0.2%Eu_2_L_3_	52.73	3.95	55.92	98.57	0.800	36.84
BPA.DA-NVP@0.5%Eu_2_L_3_	31.45	3.56	32.73	61.09 ^1^ 73.98 ^2^	0.748 ^1^ 0.763 ^2^	43.68
BPA.DA-NVP @1%Eu_2_L_3_	28.60	2.96	29.89	50.11 ^1^ 70.74 ^2^	0.705 ^1^ 0.648 ^2^	52.09
BPA.DA-NVP@1%Eu(NO_3_)_3_	85.58	3.94	86.51	134.05	0.492	42.24
BPA.DA-NVP@1%H_2_L	38.88	3.37	41.46	70.21 ^1^ 99.44 ^2^	0.437 ^1^ 0.578 ^2^	56.30

^1,2^—two maxima of the tan δ_max_.

**Table 5:** Flexural strength data for obtained materials.

Sample	Stress at Break (MPa)	Relative Elongate at Break (%)	Young’s Modulus (GPa)
	Avg	Avg	Avg
BPA.DA-NVP	111.5 ± 0.5	3.48 ± 0.05	3.66 ± 0.08
BPA.DA-NVP@0.1%Eu_2_L_3_	104.8 ± 4.8	3.05 ± 0.14	3.79 ± 0.09
BPA.DA-NVP@0.2%Eu_2_L_3_	107.76 ± 4.47	3.34 ± 0.36	3.90 ± 0.29
BPA.DA-NVP@0.5%Eu_2_L_3_	75.77 ± 4.91	3.95 ± 0.40	2.98 ± 0.18
BPA.DA-NVP@1%Eu_2_L_3_	14.55 ± 0.26	>7 * ± 1.0	0.34 ± 0.02
BPA.DA-NVP@1%Eu(NO_3_)_3_	97.17 ± 1.52	2.67 ± 0.22	3.81 ± 0.08
BPA.DA-NVP@1%H_2_L	61.31 ± 3.92	3.75 ± 0.34	2.48 ± 0.30

*—the sample did not break due to high elasticity.

**Table 6:** Data of hardness test of the synthesized samples.

Sample	Hardness (°Sh) (D Scale)	
	Samples before DMA	Samples after DMA
BPA.DA-NVP	81.7 ± 1.1	83.0 ± 1.1
BPA.DA-NVP@0.1%Eu_2_L_3_	81.0 ± 0.4	83.2 ± 1.5
BPA.DA-NVP@0.2%Eu_2_L_3_	80.7 ± 1.3	80.7 ± 1.0
BPA.DA-NVP@0.5%Eu_2_L_3_	59.5 ± 1.5	59.2 ± 1.5
BPA.DA-NVP@1%Eu_2_L_3_	42.0 ± 1.5	44.2 ± 0.8
BPA.DA-NVP@2%Eu_2_L_3_	19.8 ± 1.9	- *
BPA.DA-NVP@1%Eu(NO_3_)_3_	81.0 ± 0.3	84.2 ± 1.5
BPA.DA-NVP@1%H_2_L	77.8 ± 0.7	76.0 ± 1.5

*—no measurement was made due to the inappropriate form of the sample.

## Data Availability

The data underlying this article will be shared on reasonable request from the corresponding authors.

## Supplementary Materials

The following supporting information can be downloaded at: https://www.mdpi.com/article/10.3390/ma16196509/s1, Figure S1: ATR/IR spectra of polymeric BPA.DA–NVP matrix and hybrid BPA.DA-NVP@0.1/0.2/0.5/1/2%Eu_2_L_3 materials_; Figure S2: ATR/IR spectra of polymeric BPA.DA–NVP matrix and hybrid BPA.DA-NVP@1%Eu(NO_3_)_3_, BPA.DA-NVP@1%H_2_L, BPA.DA-NVP@1%Eu_2_L_3 materials_; Figure S3: emission spectra of BPA.DA–NVP@1%H_2_L in matrix and free matrix (BPA.DA–NVP), λ_ex_ = 340 nm; Figure S4: comparison of emission intensity of materials with Eu(III) complex and sample of Eu(NO_3_)_3_ in BPA-DA-NVP matrix, λ_ex_ = 393 nm; Figure S5: luminescence decay curves; Figure S6: DSC curves of polymeric BPA.DA–NVP matrix and hybrid BPA.DA-NVP@0.1/0.2/0.5/1/2%Eu_2_L_3 materials_; Figure S7: DSC curves of polymeric BPA.DA–NVP matrix and hybrid BPA.DA-NVP@1%Eu(NO_3_)_3_, BPA.DA-NVP@1%H_2_L, BPA.DA-NVP@1%Eu_2_L_3 materials_; Figure S8: FTIR spectra of the 2-pyrrolidone as gaseous product in the decomposition process; Figure S9: FTIR spectra of the 4-methylphenol as gaseous product in the decomposition process; Figure S10: FTIR spectra of the 4-propylphenol as gaseous product in the decomposition process; Figure S11: FTIR spectra of the Bisphenol A as gaseous product in the decomposition process; Figure S12: FTIR spectra of the phenol as gaseous product in the decomposition process; Table S1: amounts of substrates used for synthesis of materials.
