# Supplementary material for: Luminescent Hybrid BPA.DA-NVP@Eu2L3 Materials: In Situ Synthesis, Spectroscopic, Thermal, and Mechanical Characterization
Source: Materials (Basel). 2023 Sep 30;16(19):6509. doi: 10.3390/ma16196509 (PMC10573574; doi:10.3390/ma16196509)
Supplement: Supplementary file 1 [file materials-16-06509-s001.zip › materials-2611744-supplementary.pdf]

Luminescent Hybrid BPA.DA-NVP@Eu_2L_3 Materials: In Situ Synthesis, Spectroscopic, Thermal, and Mechanical Characterization

Dmytro Vlasyuk [1,*], Renata Łyszczek [1,*], Beata Podkościelna [2], Andrzej Puszka [2], Zbigniew Hnatejko [3], Marek Stankevič [4] and Halina Głuchowska [1]

[1] Department of General and Coordination Chemistry and Crystallography, Faculty of Chemistry, Institute of Chemical Sciences, Maria Curie-Skłodowska University, M. C. Skłodowskiej Sq. 2, 20-031 Lublin, Poland; halina.gluchowska@mail.umcs.pl

[2] Department of Polymer Chemistry, Faculty of Chemistry, Institute of Chemical Sciences, Maria
Curie-Skłodowska University, Gliniana 33, 20-614 Lublin, Poland; beata.podkoscielna@mail.umcs.pl (B.P.); andrzej.puszka@mail.umcs.pl (A.P.)

[3] Department of Rare Earths, Faculty of Chemistry, Adam Mickiewicz University in Poznań, Uniwersytetu Poznańskiego 8, 61-614 Poznań, Poland; zbigniew.hnatejko@amu.edu.pl

[4] Department of Organic Chemistry, Faculty of Chemistry, Institute of Chemical Sciences, Marie
Curie-Skłodowska University, Gliniana 33, 20-614 Lublin, Poland; marek.stankevic@mail.umcs.pl

* Correspondence: dmytro.vlasyuk@mail.umcs.pl (D.V.); renata.lyszczek@mail.umcs.pl (R.Ł.)

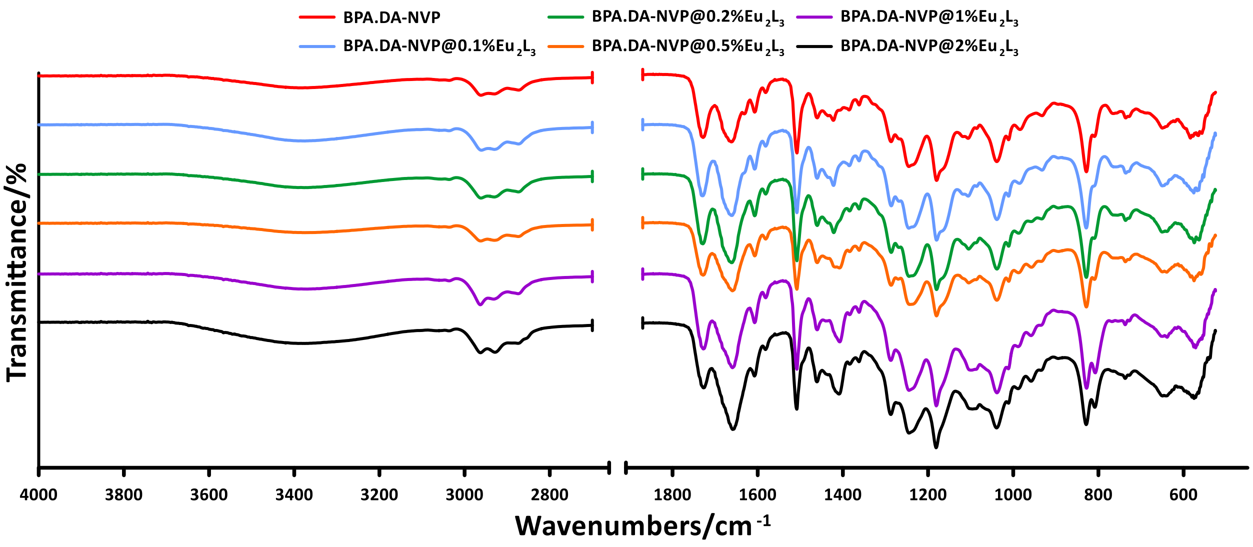

Figure S1. ATR/IR spectra of: polymeric matrix BPA.DA–NVP and hybrid materials BPA.DA-NVP@0.1/0.2/0.5/1/2%Eu_2L_3.

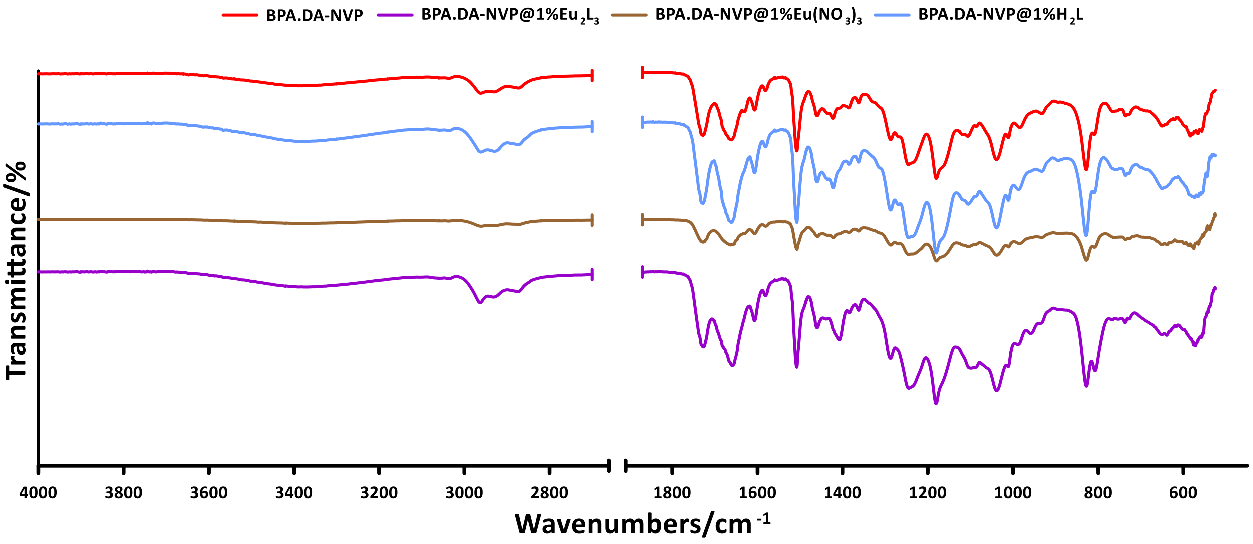

Figure S2. ATR/IR spectra of: polymeric matrix BPA.DA–NVP and hybrid materials BPA.DA-NVP@1%$Eu(NO_3)_3$, BPA.DA-NVP@1%H_2L, BPA.DA-NVP@1%Eu_2L_3.

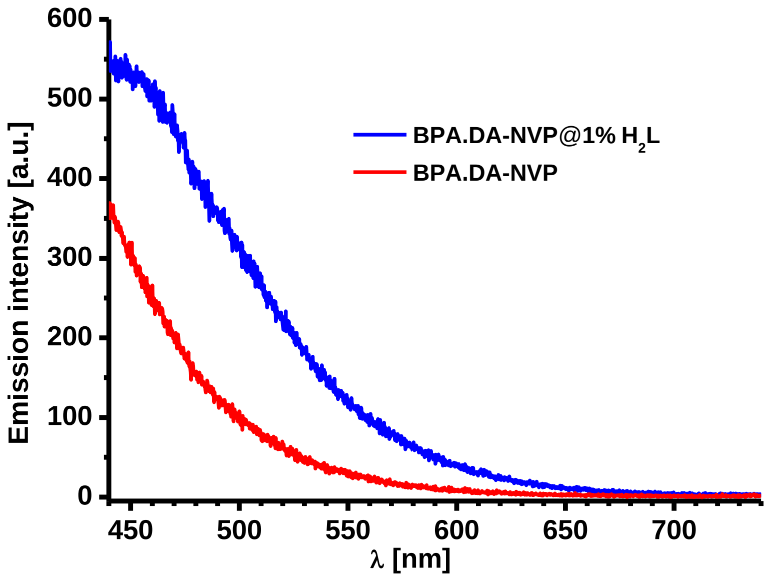

Figure S3. Emission spectra of BPA.DA–NVP@1%H_2L in matrix and free matrix (BPA.DA–NVP), λ_{ex} =340 nm

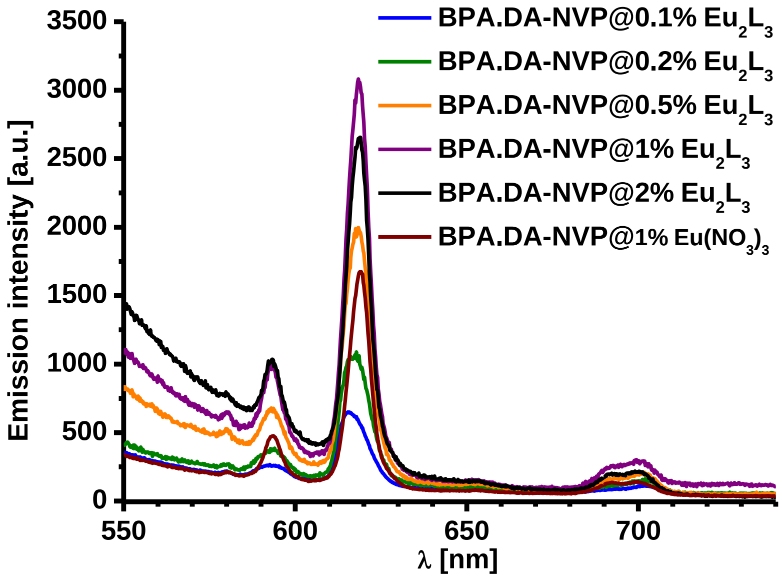

Figure S4. Comparison of emission intensity of materials with Eu(III) complex and sample of $Eu(NO_3)_3$ in BPA-DA-NVP matrix, λ_{ex}=393 nm.

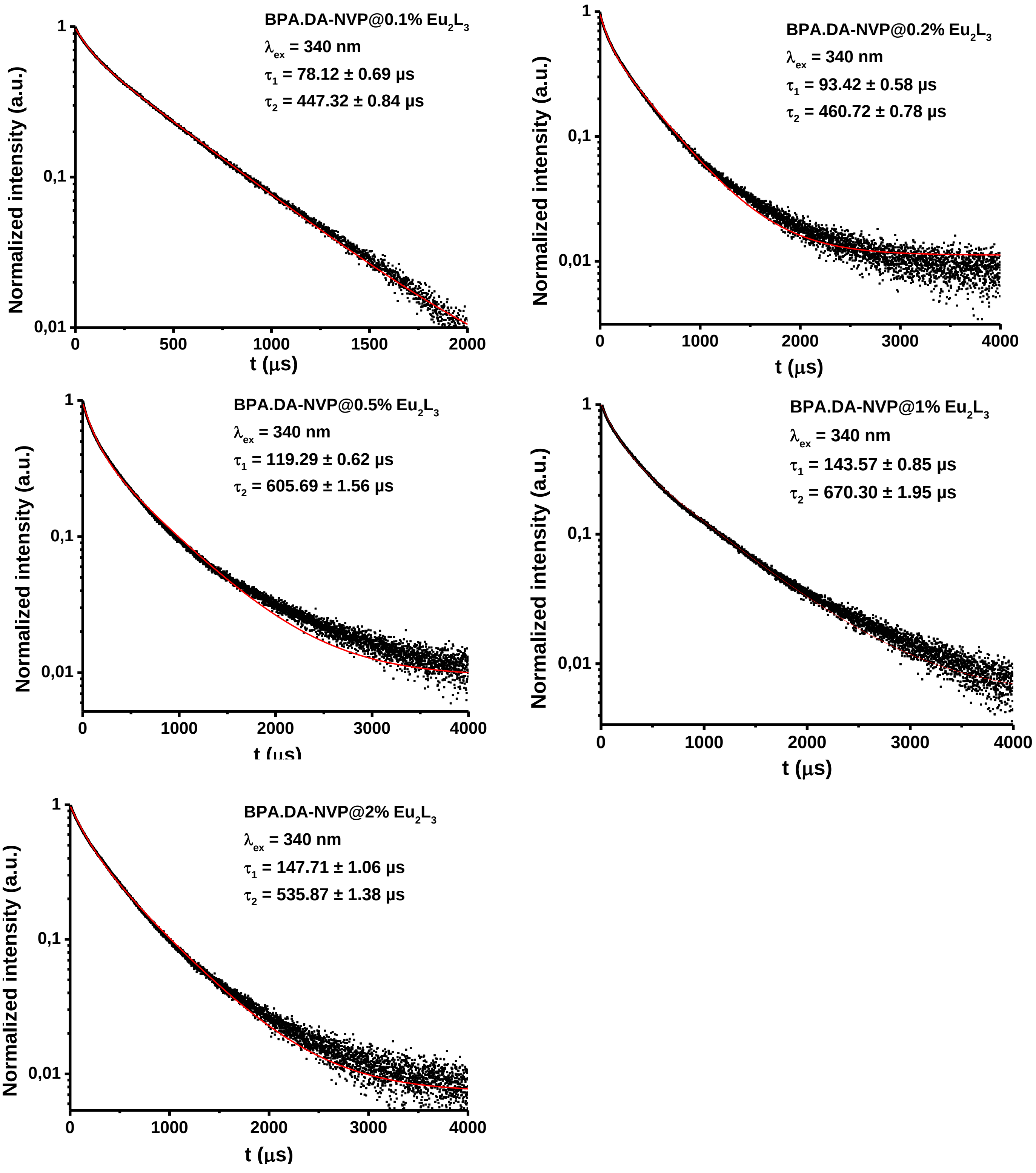

Figure S5. Luminescence decay curves.

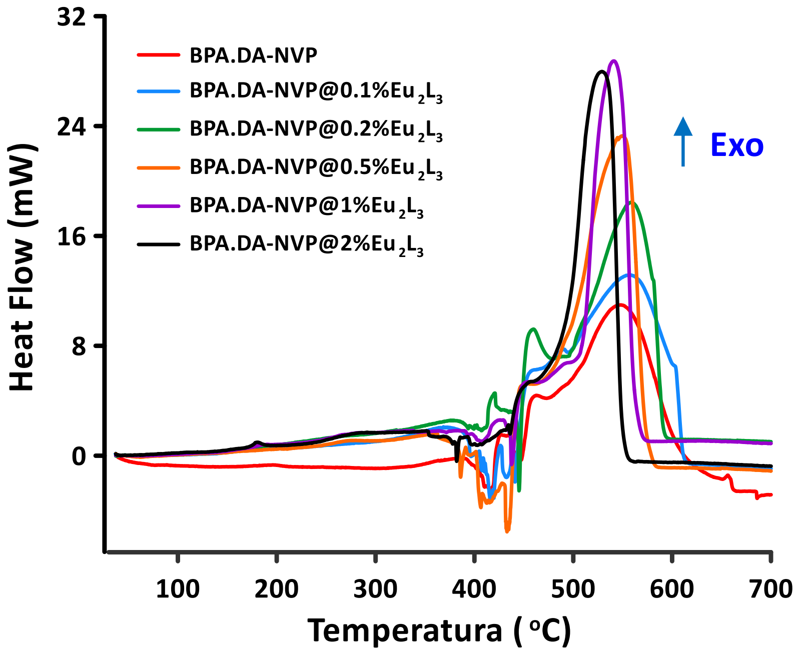

Figure S6. DSC curves of: polymeric matrix BPA.DA–NVP and hybrid materials BPA.DA-NVP@0.1/0.2/0.5/1/2%Eu_2L_3.

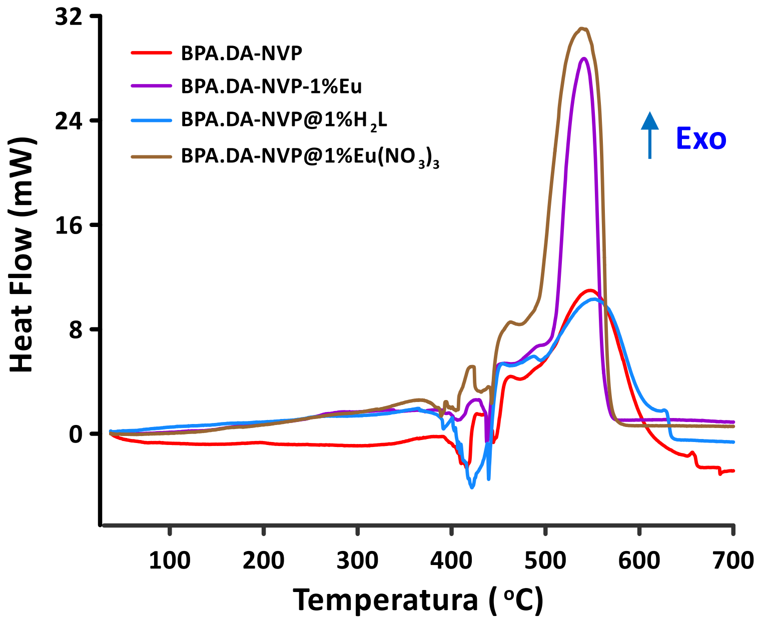

Figure S7. DSC curves of: polymeric matrix BPA.DA–NVP and hybrid materials BPA.DA-NVP@1%$Eu(NO_3)_3$, BPA.DA-NVP@1%H_2L, BPA.DA-NVP@1%Eu_2L_3.

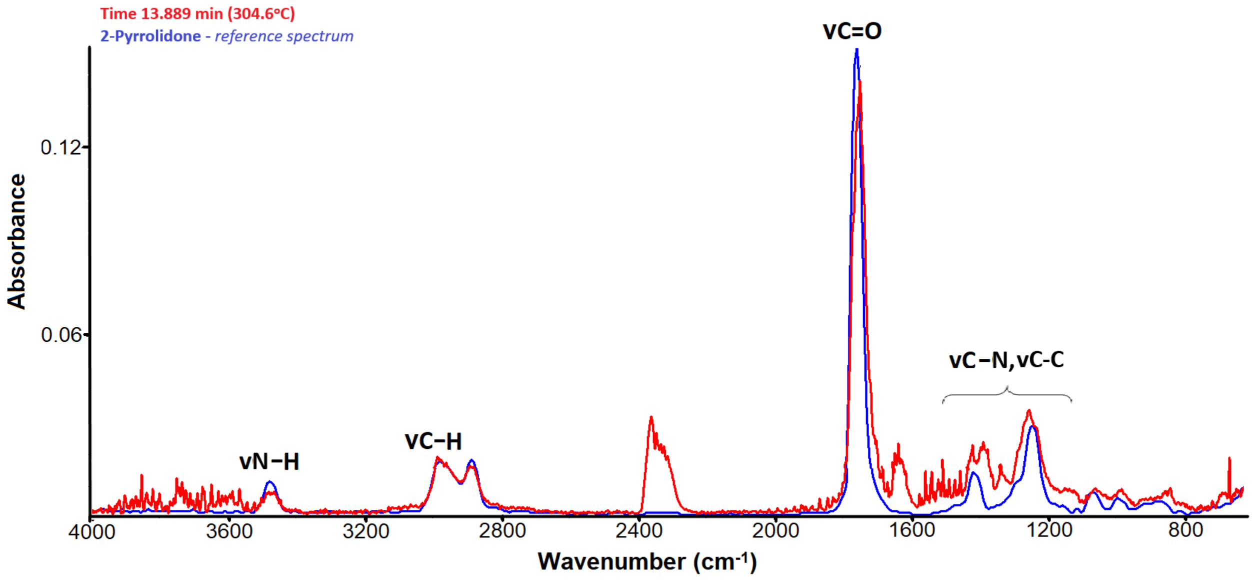

Figure S8. FTIR spectra of the 2-pyrrolidone as gaseous product in the decomposition process.

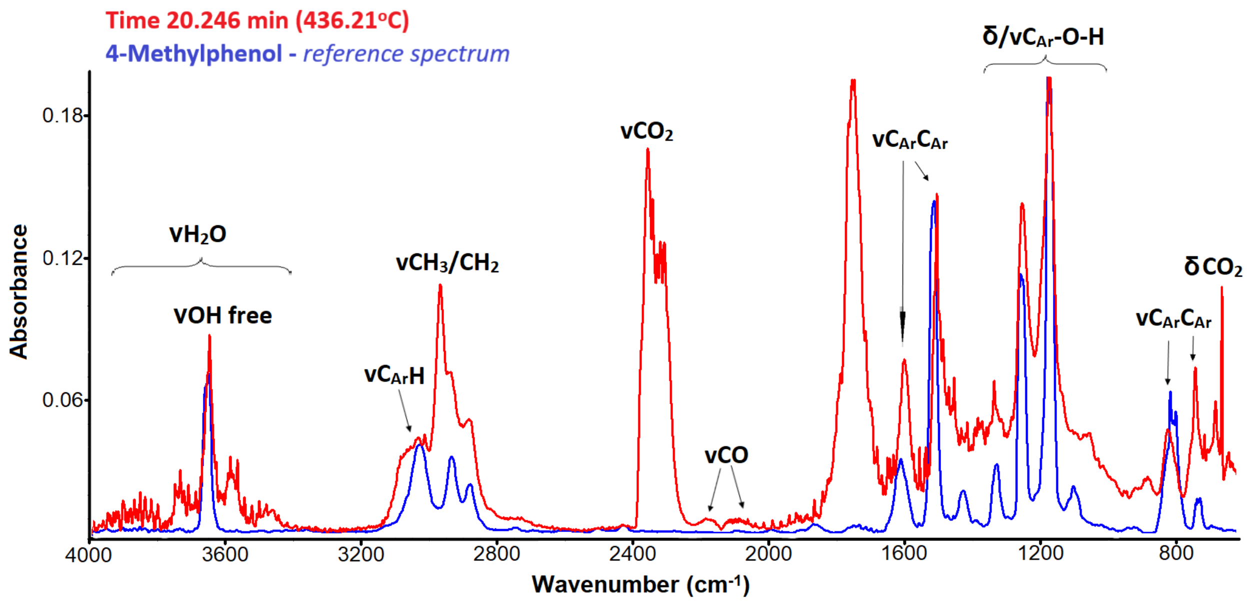

Figure S9. FTIR spectra of the 4-methylphenol as gaseous product in the decomposition process.

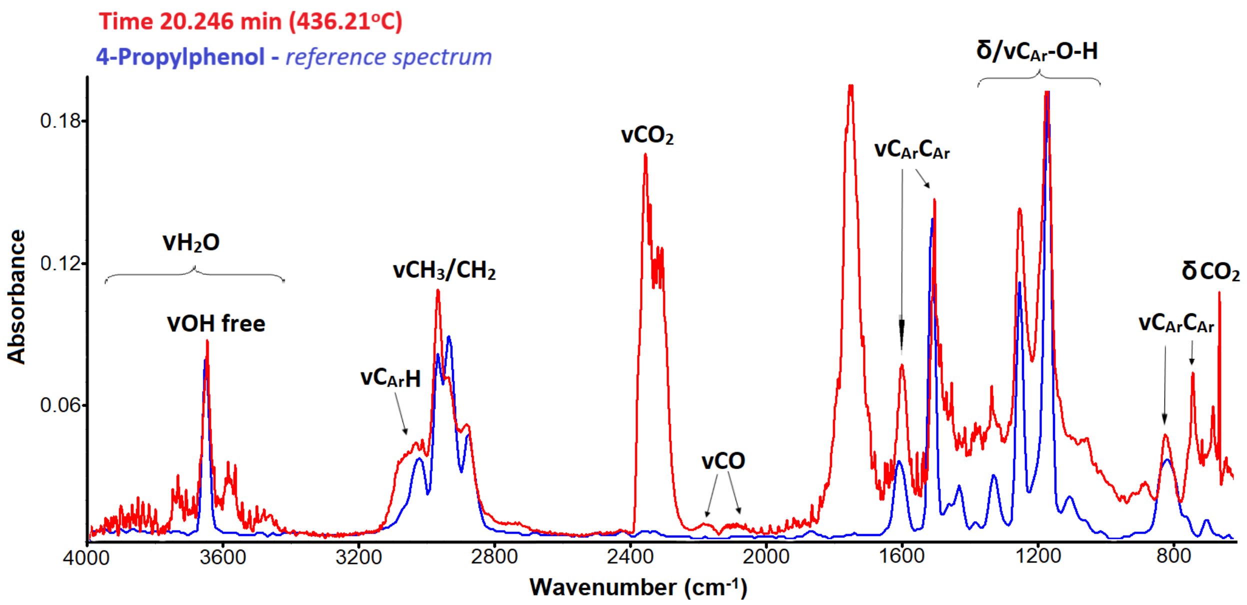

Figure S10. FTIR spectra of the 4-propylphenol as gaseous product in the decomposition process.

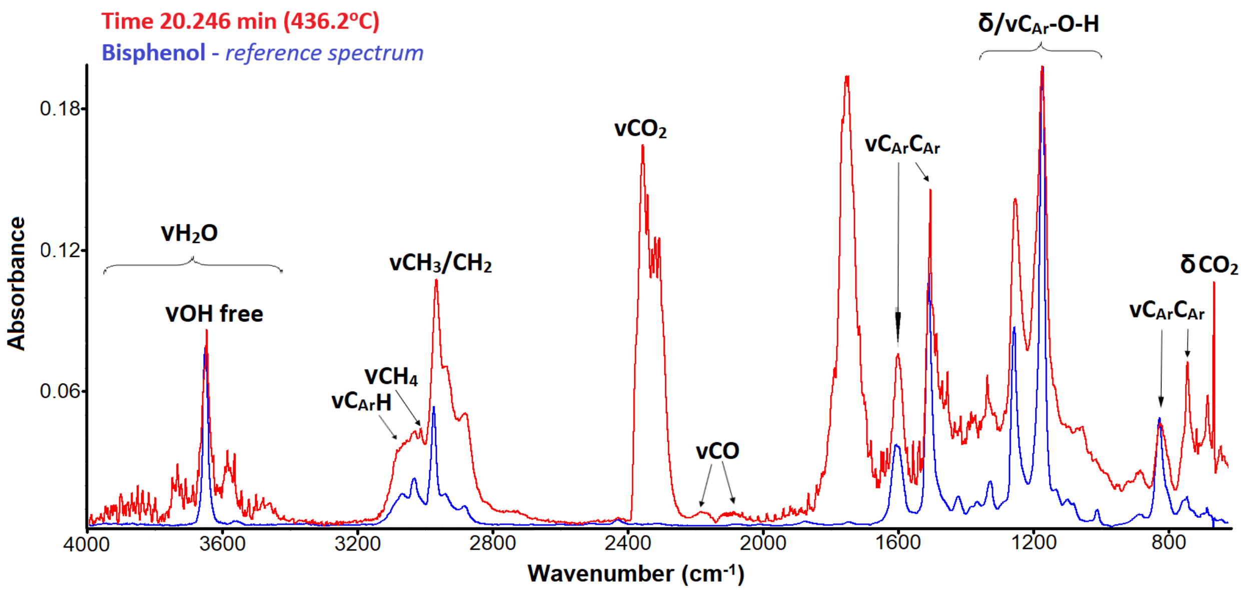

Figure S11. FTIR spectra of the Bisphenol A as gaseous product in the decomposition process.

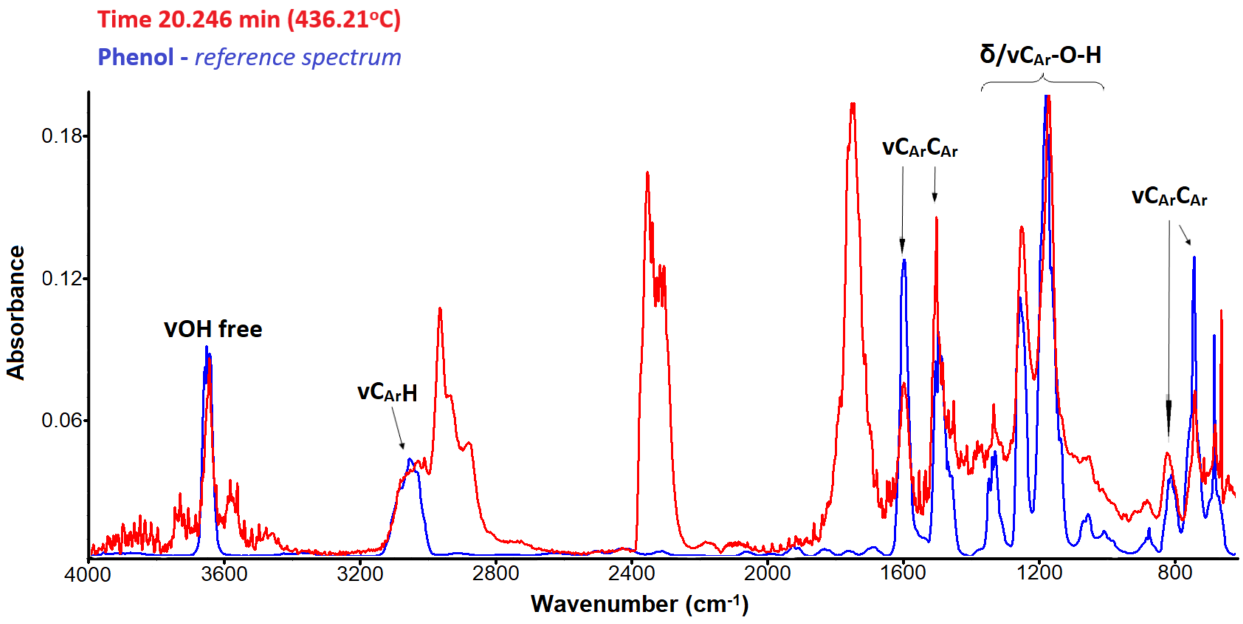

Figure S12. FTIR spectra of the phenol as gaseous product in the decomposition process.

Table S1. Amounts substrates used for materials synthesis.

Material	$Eu(NO_3)_3$ (g)	H_2L (g)	Eu_2L_3 (g)	BPA.DA (g)	NVP (g)	Irgacure 651 (g)
**BPA.DA-NVP**	-	-	-	5.6	2.4	0.08
**BPA.DA-NVP@1%Eu(NO$_3$)$_3$**	0.08	-	-	5.6	2.4	0.08
**BPA.DA-NVP@1%H$_2$L**	-	0.08	-	5.6	2.4	0.08
**BPA.DA-NVP@0.1%Eu$_2$L$_3$**	0.0062	0.0052	0.008	5.6	2.4	0.08
**BPA.DA-NVP@0.2%Eu$_2$L$_3$**	0.0125	0.0105	0.016	5.6	2.4	0.08
**BPA.DA-NVP@0.5%Eu$_2$L$_3$**	0.0313	0.0262	0.04	5.6	2.4	0.08
**BPA.DA-NVP@1%Eu$_2$L$_3$**	0.0625	0.0525	0.08	5.6	2.4	0.08
**BPA.DA-NVP@2%Eu$_2$L$_3$**	0.1251	0.1052	0.16	5.6	2.4	0.08
